# Polymorphisms of Homologous Recombination *RAD51*, *RAD51B*, *XRCC2*, and *XRCC3* Genes and the Risk of Prostate Cancer

**DOI:** 10.1155/2015/828646

**Published:** 2015-08-03

**Authors:** Maria Nowacka-Zawisza, Ewelina Wiśnik, Andrzej Wasilewski, Milena Skowrońska, Ewa Forma, Magdalena Bryś, Waldemar Różański, Wanda M. Krajewska

**Affiliations:** ^1^Department of Cytobiochemistry, Faculty of Biology and Environmental Protection, University of Lodz, 90-236 Lodz, Poland; ^2^Department of Biophysics of Environmental Pollution, Faculty of Biology and Environmental Protection, University of Lodz, 90-236 Lodz, Poland; ^3^Department of Biochemistry, Faculty of Medicine, Medical University of Lodz, 92-215 Lodz, Poland; ^4^2nd Department of Urology, Faculty of Biomedical Sciences and Postgraduate Training, Medical University of Lodz, 93-513 Lodz, Poland

## Abstract

Genetic polymorphisms in DNA repair genes may induce individual variations in DNA repair capacity, which may in turn contribute to the risk of cancer developing. Homologous recombination repair (HRR) plays a critical role in maintaining chromosomal integrity and protecting against carcinogenic factors. The aim of the present study was to evaluate the relationship between prostate cancer risk and the presence of single nucleotide polymorphisms (SNPs) in the genes involved in HRR, that is, *RAD51* (rs1801320 and rs1801321), *RAD51B* (rs10483813 and rs3784099), *XRCC2* (rs3218536), and *XRCC3* (rs861539). Polymorphisms were analyzed by PCR-RFLP and Real-Time PCR in 101 patients with prostate adenocarcinoma and 216 age- and sex-matched controls. A significant relationship was detected between the *RAD51* gene rs1801320 polymorphism and increased prostate cancer risk. Our results indicate that the *RAD51* gene rs1801320 polymorphism may contribute to prostate cancer susceptibility in Poland.

## 1. Introduction

Prostate cancer (PC) is the second most commonly diagnosed malignant disease in men and the sixth leading cause of cancer-related death among men worldwide, with an estimated 10948 (14.3%) registered new cases and 4199 (8%) deaths in 2012 in Poland [1]. Although prostate cancer is one of the most common cancers in men, the genetic defects underlying its pathogenesis remain poorly understood. While being over 65 years and the presence of a family history are the strongest risk factors for prostate cancer, ethnicity has also been shown to be a risk factor, with the lowest incidence rates of prostate cancer being observed in Asian men, particularly in India, China, and Japan. Higher incidence rates are seen in black men. The risk of developing prostate cancer is thought to be 1.3–2.0 times higher in African-American than Caucasian men [2].

Genetic defects in DNA repair and DNA damage response genes often lead to an increase of cancer incidence. The most deleterious form of DNA damage is the DNA double-strand break (DSB), which can be formed by free radicals derived from the metabolism, ionizing radiation, or DNA cross-linking agents or which can naturally occur during DNA replication. Unrepaired DSBs lead to mutations, rearrangements, and/or loss of chromosomes, causing genome instability and the development of tumors or cell death. In order to maintain genetic stability, DNA double-strand breaks must be repaired by homologous recombination or nonhomologous end-joining. The key protein in homologous recombination is RAD51, which displaces replication protein A (RPA) and forms a helical nucleofilament on the exposed single-stranded DNA flanking the DSB. This nucleofilament performs a homology search for repair template and then catalyses DNA strand invasion [3, 4].

RAD51 is a homologue of the RecA protein and contains 339 amino acids. The* RAD51* gene is located at human chromosome 15q15.1 and is highly polymorphic. Five RAD51 paralogs, that is, RAD51B, RAD51C, RAD51D, XRCC2, and XRCC3, have been identified in the human genome. They are key components of homologous recombination, and their loss can result in the development of genetic instability. The RAD51 paralogs form two major complexes, RAD51B-RAD51C-RAD51D-XRCC2 (BCDX2) and RAD51C-XRCC3 (CX3), as well as two subcomplexes, RAD51B-RAD51C (BC) and RAD51D-XRCC2 (DX2). The CX3 complex is known to catalyze strand exchange* in vitro*. Similarly, the BC subcomplex of the BCDX2 complex has been reported to have* in vitro* RAD51 mediator activity and the DX2 subcomplex has been reported to have strand exchange activity. However, the specific activities of the RAD51 paralog complexes and subcomplexes have not been defined* in vivo* [5–7].

The genetic variations of* RAD51* and its paralogs may contribute to the development of cancer, as has been shown in the case of breast, ovarian, endometrial, colorectal, and head and neck cancer and acute leukemia [8–11].

The aim of the work was to evaluate the significance of common genetic variation in four genes involved in DNA double-strand break repair* via* homologous recombination, that is,* RAD51*,* RAD51B*,* XRCC2*, and* XRCC3*, in prostate cancer susceptibility, tagging six most widely studied single nucleotide polymorphisms (SNPs) in these genes. The association between the presence of polymorphisms rs1801320 and rs1801321 of* RAD51*, rs10483813 and rs3784099 of* RAD51B*, rs3218536 of* XRCC2*, and rs861539 of* XRCC3* and the risk of prostate cancer was examined.

## 2. Material and Methods

### 2.1. Study Subjects

The study included 101 men with prostate adenocarcinoma and 216 sex- and age-matched persons who have not been diagnosed with cancer to serve as a control group. Peripheral blood from the patients with prostate adenocarcinoma was collected at the 2nd Department of Urology, Medical University of Lodz, Poland, between October 2009 and December 2011. Peripheral blood from the control group was obtained from the Maria Sklodowska-Curie Memorial Hospital, Zgierz, Poland. Blood samples were collected on EDTA and frozen. The clinical characteristics of the prostate cancer patients and controls are presented in Table 1. None of the patients underwent any anticancer treatment. The samples were obtained in accordance with guidelines concerning ethical and legal requirements. Informed consent was obtained from patients, and the studies were approved by the Independent Ethical Committees of the Medical University of Lodz, Poland (RNN/59/09/KE), and the University of Lodz, Poland (KBBN-UŁ/II/25/2012).

### 2.2. DNA Isolation

DNA was isolated from the blood samples from the prostate adenocarcinoma patients and controls using an AxyPrep Blood Genomic DNA Miniprep Kit (Axygen, USA) and the phenol-chloroform method. DNA purity and quantity were estimated by UV-spectroscopy (Eppendorf BioPhotometer TM Plus, Eppendorf, Germany). DNA purity was determined by the 260/280 nm absorbance ratio, with a value between 1.8 and 2.0 being acceptable.

### 2.3. Genotyping

Single nucleotide polymorphisms (SNPs) of the* RAD51* (rs1801320 and rs1801321),* RAD51B* (rs10483813 and rs3784099),* XRCC2* (rs3218536), and* XRCC3* (rs861539) genes were analyzed (Table 2).

The genotyping of rs1801320, rs3218536, and rs861539 polymorphisms was determined by PCR-RFLP. The primers and PCR conditions for the polymorphic sites of these genes are shown in Table 2. The PCR was run in 10 *μ*L reactions containing 10 ng of genomic DNA, 0.2 mM of each primer, 2.5 mM MgCl_2_, 1 mM deoxyribonucleotide triphosphates (dNTPs), 3 U HOT FIREPol DNA polymerase, and 1x Solis BioDyne buffer B1. The primers were synthesized by Sigma-Aldrich (USA), and PCR reagents were obtained from Solis BioDyne (Estonia) and Applied Biosystems (USA). Thermal cycling was performed as follows: initial activation at 95°C for 12 min, followed by 30 amplification cycles consisting of denaturation at 95°C for 30 s, annealing at 64°C (rs3218536, rs861539) or 65°C (rs1801320) for 30 s, and extension at 72°C for 1 min, followed by a final extension at 72°C for 10 min.

**Figure 1:**
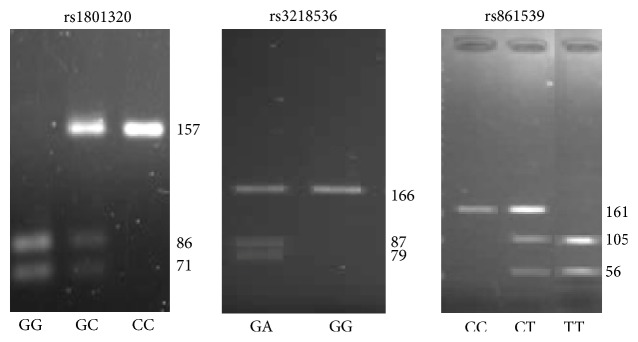
Genotyping of rs1801320, rs3218536, and rs861539 polymorphisms by PCR-RFLP.

To genotype the rs1801320, rs3218536, and rs861539 polymorphisms, 10 *μ*L of each PCR product was digested with either 2 U of* MvaI* (*BstNI*) (Thermo Scientific, USA), 1 U of* SexAI* (New England Biolabs Inc., USA), or 0.5 units of* NlaIII* (New England Biolabs Inc., USA), respectively, for 16 h at 37°C. The genotypes were determined by running the digested products in 3% agarose gel with ethidium bromide (1 *μ*L/mL) for UV visualization. The products for each genotype of the tested genes are shown in Table 3. Examples of the obtained restriction patterns are presented in Figure 1.

The rs1801321, rs10483813, and rs3784099 polymorphisms were genotyped by Real-Time PCR (Mastercycler ep realplex, Eppendorf USA) using TaqMan SNP Genotyping Assays (Applied Biosystems). The PCR amplification was performed according to the manufacturer's recommendations in 10 *μ*L reactions containing 10 ng of genomic DNA, 0.25 *μ*L SNP Genotyping Assay Mix (40x) (rs1801321 Assay ID: C_7482700_10; rs10483813 Assay ID: C_2564845_10; rs3784099 Assay ID: C_27481679_10), and 5 *μ*L TaqMan Universal PCR Master Mix (2x). The amplification conditions consisted of initial AmpliTaq Gold 1 activation at 95°C for 10 min, followed by 40 amplification cycles consisting of denaturation at 95°C for 15 s and annealing/extension at 60°C for 1 min.

### 2.4. Statistical Analysis

The chi-square test was used to analyze the Hardy-Weinberg frequencies for the* RAD51*,* RAD51B*,* XRCC2*, and* XRCC3* genotypes in the control and patients populations. The differences in genotype and allele frequency between prostate cancer patients and controls were also evaluated with the chi-square test. The odds ratio (OR) and corresponding 95% confidence intervals (CI) were determined through unconditional multiple logistic regression. Statistical analysis was carried out using Statistica version 9.0 (StatSoft, Poland).

## 3. Results

The study comprised 101 prostate cancer cases and 216 sex- and age-matched controls. The genotypes of six genetic variants of the* RAD51*,* RAD51B*,* XRCC2*, and* XRCC3* genes were determined. The genotypes and allele frequencies for each polymorphism of the studied genes are summarized in Table 4. The observed genotype frequencies were found to be consistent with the Hardy-Weinberg equilibrium at all studied polymorphic loci, except for the* RAD51* polymorphism. No statistically significant differences were found between prostate cancer patients and cancer-free controls with regard to the distribution of genotypes and alleles, apart from the rs1801320 polymorphism of the* RAD51* gene.

The genotype frequencies for each polymorphism, in both the prostate cancer group and the control group, were analyzed using a logistic regression model (Table 5). Among the six polymorphisms examined, the rs1801320* RAD51* gene polymorphism was found to be significantly associated with prostate cancer susceptibility. The rs1801320* RAD51* gene polymorphism was verified by sequencing analysis with BigDye Terminator Cycle Sequencing Ready Reaction Kits version 1.1 (see Supplementary Material available online at http://dx.doi.org/10.1155/2015/828646). Heterozygous (OR = 1.90) and homozygous (OR = 2.98) C genotype variants and C allele (OR = 2.02, *P* < 0.01) appeared to increase prostate cancer risk.

The association between the C allele of* RAD51* gene rs1801320 polymorphism and the age and PSAT serum level of the cancer patients was analyzed. No statistically significant relationship was found between the presence of the C allele for rs1801320 and either age or PSAT serum level (Table 6).

## 4. Discussion

Despite the high incidence of prostate cancer, the exact cause of its development is not known. It is assumed that, as in the case of other cancers, prostate cancer occurs as a result of the interaction between environmental factors and genetic predisposition. The standard biomarker associated with prostate pathology is prostate specific antigen (PSA). PSA is a kallikrein-like serine protease secreted by the epithelial cells of the prostate and encoded by an androgen-responsive gene (19q 13.3–13.4). The major role of PSA is the liquefaction of human semen by its proteolytic activity. However, because of the low specificity of PSA, unnecessary biopsies and mistaken diagnoses frequently occur. As prostate cancer has a variety of features, prognosis following diagnosis is greatly variable. Hence, there is a need for new biomarkers to improve clinical management of prostate cancer [12].

DNA is replicated with extremely high fidelity in normal cells, with a mutation rate of 10^−10^ per base pair per cell division. DNA damage typically occurs through exposure to genotoxic chemicals, ultraviolet and ionizing radiation, failures in normal cellular DNA processing and replication events (stalled replication forks), and spontaneous DNA-damaging events. These processes contribute to oxidation, alkylation, cross-linking, and dimerization and cause strand breaks in DNA, whose repair is essential for maintaining the integrity of the genome and preventing cancer. The DNA double-strand break is the most lethal form of DNA damage, as it can lead to significant DNA damage by multiple genomic changes, including translocation, deletion, and amplification, resulting in heritable cellular genomic instability that can lead to malignancy [12, 13].

The repair of a double-strand DNA break is performed by homologous recombination. This process is promoted by recombinase RAD51, which catalyzes the key reactions that typify HRR, that is, homology search and DNA strand invasion. Several RAD family members, including its paralogs RAD51B, XRCC2, and XRCC3, assist RAD51 in this process [14–16].

There is a growing body of evidence, which suggests that polymorphic variants of genes involved in DNA repair could modulate DNA repair capacity and thus have a great impact on genomic stability and cancer prevention. Human* RAD51* and its paralogs* RAD51B*,* XRCC2*, and* XRCC3* are highly polymorphic. The* RAD51* polymorphisms c. -98G>C (rs1801320; 135G>C) and c. -61G>T (rs1801321; 172G>T) are two of the most common polymorphisms and are located at 5′UTR. Although the functional consequence of the c. -98G>C polymorphism is unknown, it is speculated that because it alters a CpG island pattern in the promoter region, it may regulate the expression of* RAD51* and its mRNA levels. The c. -61G>T is located in a binding site for the transcription factor P300/CBP. Current models suggest that the binding of P300/CBP cofactors to transcription factor activation domains positions histone acetyltransferases near specific nucleosomes in the promoter regions of the target gene. In contrast to the c. -61T allele, the c. -61G allele does not form a binding site for cis-transcriptional elements for P300/CBP. Thus, the presence of the T allele results in a greater effect on* RAD51* gene expression [17–19].

It was found that the* RAD51* polymorphism rs1801320 is associated with an elevated risk of breast [20–22] and triple-negative breast cancer [23] and ovarian [24, 25], endometrial [26–28], colorectal [9, 29, 30], gastric [31], and head and neck cancer [32, 33] and myelodysplastic syndrome [34], as well as keratoconus and Fuchs endothelial corneal dystrophy [35]. The significance of the* RAD51* polymorphism rs1801320 has been best characterized in patients with breast cancer, especially in carriers of* BRCA2* gene mutations. Antoniou et al. [17] report that the risk of developing breast cancer increases in the case of a polymorphic variant c. -98G>C and the presence of mutations in the* BRCA2* gene. No correlations of this polymorphism were found in carriers of* BRCA1* gene mutations. These results are confirmed by numerous studies [36–39]. However, on the contrary, Wang et al. [40] observe that the* RAD51* gene rs1801320 polymorphism reduces the risk of developing ovarian cancer in carriers of the* BRCA2* mutations. In addition, Ribeiro Junior et al. [41] indicate that the rs1801320 polymorphism of* RAD51* gene is associated with a decreased risk of developing myelodysplastic syndrome. No correlation was found between the presence of the* RAD51* rs1801321 polymorphism and the risk of developing endometrial and ovarian cancer [28, 42]. However, the variant c. -61G>T in the* RAD51* gene seems to affect the development of breast and triple-negative breast cancer, acute myeloid leukemia, and head and neck cancer [32, 43–46].

The* RAD51B* gene encodes one of five RAD51 paralogs, which play an important role in DNA repair through homologous recombination. RAD51B-deficient chicken B lymphocyte DT40 cells have been observed to impair homologous recombination and are sensitive to cross-linking agents. It has been suggested that RAD51B promotes the assembly of the RAD51 nucleoprotein filaments during HRR. Inactivation of RAD51B by translocation between chromosomes 12 and 14 is a frequent finding in uterine leiomyoma, supporting a role for the inactivation of RAD51B in tumorigenesis [47]. In the case of the* RAD51B* gene, a correlation has been demonstrated between the presence of the rs3784099 polymorphism and the risk of breast cancer [48]. The relationship between the* RAD51B* gene rs10483813 polymorphic variants and risk of triple-negative breast cancer and age-related macular degeneration has also been indicated [49, 50].

The* XRCC2* and* XRCC3* genes are recognized as essential parts of the homologous recombination repair pathway. They are required for correct chromosome segregation and apoptotic response to DSB [51, 52]. A meta-analysis of data from available literature revealed no direct relationship between polymorphic variants rs3218536 of the* XRCC2* gene and the risk of breast cancer [53]. However, the* XRCC2* Arg188His polymorphism (c. 563G>A; rs3218536) is suggested to modify the risk of breast cancer, including triple-negative breast cancer [20, 54, 55]. A correlation was found between the prevalence of the* XRCC2* gene rs3218536 polymorphism and morbidity of triple-negative breast cancer in the female population in Poland [55]. The* XRCC2* gene rs3218536 polymorphism has been found to possibly increase the risk of developing cervical cancer and breast cancer in women in Pakistan [11, 56]. In contrast, no direct relation was observed between the occurrence of rs3218536 variant of the* XRCC2* gene and the incidence of lung cancer or sporadic colorectal cancer [57, 58]. Similarly, no correlation has been found with hereditary colon cancer, known as Lynch syndrome [59].

The Thr241Met substitution is the most thoroughly analyzed polymorphism in the* XRCC3* gene (c. 722C>T; rs861539). Although the functional relevance of the XRCC3 Thr241Met variation is unknown, some studies have reported that this polymorphism is associated with increased risk of breast cancer [20]. A meta-analysis has revealed that the* XRCC3* gene rs861539 polymorphism also increases the risk of developing hepatocellular carcinoma, as well as head and neck, bladder, and breast cancer [11, 32, 33, 60–62]. However, this polymorphism has also been shown to have a weak association with the risk of breast cancer as well as lung cancer [63, 64] and no connection with the development of ovarian, cervical, or colorectal cancer or renal cell carcinoma [9, 65–67]. No direct relationship has been found between the* XRCC3* gene rs861539 polymorphism and the risk of leukemia in the general population. However, studies performed in Asian populations have implicated it in the risk of incidence of leukemia [68].

The results of the present study show that the* RAD51* gene rs1801320 polymorphism doubles the risk of prostate cancer in the studied population. Statistically significant differences in allele distribution were identified between the control group and prostate cancer patients (*P* < 0.05). Dhillon et al. [69] do not report any such correlation in an Australian population. Hence, the* RAD51* gene rs1801320 polymorphism may act as an independent biomarker of prostate cancer risk in Polish population.

No associations were found between the polymorphic variants of the* RAD51B*,* XRCC2*, and* XRCC3* genes evaluated in the present study and the susceptibility of prostate cancer. However, considering that the occurrence of cancer is the result of complex interactions between genetic changes and environmental factors, polymorphic variants of the* RAD51B*,* XRCC2*, and* XRCC3* genes other than those studied may yet have an impact on the development of prostate cancer.

## Supplementary Material

Associated with risk of prostate cancer the rs1801320 polymorphism of *RAD51* gene was verified by sequencing analysis. PCR products of each genotype were sequenced according to the manufacturer's protocol using BigDye Terminator Cycle Sequencing Ready Reaction Kits version 1.1 in ABI PRISM 377™ DNA Sequencer (Applied Biosystems). Supplementary figure has shown results of sequencing of GG, GC, CC genotypes for the rs1801320 polymorphism in *RAD51* gene.

## Figures and Tables

**Table 1:** Clinical characteristic of studied material.

	Prostate cancer patients	Control group
Age (year)		
Range	49–86	43–84
Mean ± SD	71 ± 9	63 ± 9
Median	71	63

PSAT (ng/mL)		
Range	4.01–1489	0.004–3.94
Mean ± SD	59.96 ± 182.67	1.09 ± 0.88
Median	10.57	0.90

**Table 2:** SNPs analyzed.

Gene	Polymorphism	Other names	SNP position	Chromosome	Global MAF	Method
*RAD51 *	rs1801320	c. -98G>C G135C	UTR-5, Exon	15:40695330	13.04%	PCR-RFLP
	rs1801321	c. -61G>T, G172T	UTR-5, Exon	15:40695367	26.63%	Real-Time PCR

*RAD51B *	rs10483813	c. 1037-29918T>A	Intron	14:68564567	12.49%	Real-Time PCR
	rs3784099	c. 757-8674G>A	Intron	14:68283210	37.79%	Real-Time PCR

*XRCC2 *	rs3218536	c. 563G>A, p. Arg188His	Missense	7:152648922	4.27%	PCR-RFLP

*XRCC3 *	rs861539	c. 722C>T, p. Thr241Met	Intron, Missense	14:103699416	25.07%	PCR-RFLP

**Table 3:** Details of PCR-RFLP of the studied SNPs.

SNP	Primer sequences	Annealing temp. (°C)	Product size (bp)	Enzyme	Genotype	Fragment sizes (bp)
rs1801320	(F) 5′TGGGAACTGCAACTCATCTGG3′ (R) 5′GCGCTCCTCTCTCCAGCAG3′				GG	71, 86
		65	157	*MvaI *	GC	71, 86, 157
					CC	157

rs3218536	(F) 5′CGTCAATGGAGGAGAAAGTGTG3′ (R) 5′TCGAGAGGCATGAGAAGGTT3′				GG	166
		64	166	*SexA1 *	GA	79, 87, 166
					AA	79, 87

rs861539	(F) 5′TAAGAAGGTCCCCGTACTCC3′ (R) 5′CTGCGCATCAACCAGGTGAG3′				CC	34, 161
		64	195	*NlaIII *	CT	56, 105, 161
					TT	56, 105

**Table 4:** Distribution of genotypes and allele frequency in the *RAD51*, *RAD51B*, *XRCC2*, and *XRCC3* loci among prostate cancer patients.

Gene	Genotype/allele	Prostate cancer patients (*n* = 101)	Control group (*n* = 216)	*P*
*RAD51* rs1801320	GG	66	172	**0.017**
	GC	27	37	
	CC	8	7	
	G	159	381	**0.002**
	C	43	51	
rs1801321	GG	39	67	0.392
	TG	4	8	
	TT	58	141	
	G	82	421	0.058
	T	120	290	

*RAD51B* rs10483813	TT	56	134	0.350
	TA	40	68	
	AA	5	14	
	T	152	336	0.481
	A	50	96	
rs3784099	GG	49	122	0.225
	GA	41	80	
	AA	11	14	
	G	139	324	0.102
	A	63	108	

*XRCC2* rs3218536	GG	90	196	0.648
	GA	11	20	
	AA	0	0	
	G	191	412	0.800
	A	11	20	

*XRCC3* rs861539	CC	54	119	0.776
	CT	34	75	
	TT	13	22	
	C	142	313	0.574
	T	60	119	

**Table 5:** Genotype distribution and prostate cancer risk for the *RAD51*, *RAD51B*, *XRCC2*, and *XRCC3* polymorphisms in prostate cancer patients and control group.

Genotype/allele	Prostate cancer patients (*n* = 101)	Control group (*n* = 216)	OR [95% Cl]	*P* value
rs1801320				
GG	66 (65.3)	172 (79.5)	1 [Ref.]	
GC	27 (26.7)	37 (17.3)	1.90 [1.07–3.37]	0.03
CC	8 (8.0)	7 (3.2)	2.98 [1.04–8.54]	0.04
G	159 (78.7)	381 (88.2)	1 [Ref.]	
C	43 (21.3)	51 (11.8)	2.02 [1.29–3.16]	<0.01

rs1801321				
GG	39 (38.6)	67 (31.0)	1 [Ref.]	
TG	4 (4.0)	8 (3.7)	0.86 [0.24–3.04]	1
TT	58 (57.4)	141 (65.3)	0.71 [0.43–1.16]	0.17
G	82 (40.6)	142 (32.9)	1 [Ref.]	
T	120 (59.4)	290 (67.1)	0.72 [0.51–1.01]	0.06

rs10483813				
TT	56 (55.4)	134 (62.0)	1 [Ref.]	
TA	40 (39.6)	68 (31.5)	1.41 [0.85–2.32]	0.18
AA	5 (5.0)	14 (6.5)	0.85 [0.29–2.49]	0.78
T	152 (75.2)	336 (77.8)	1 [Ref.]	
A	50 (24.8)	96 (22.2)	1.15 [0.78–1.70]	0.48

rs3784099				
GG	49 (48.5)	122 (56.5)	1 [Ref.]	
GA	41 (40.6)	80 (37.0)	1.28 [0.77–2.11]	0.34
AA	11 (10.9)	14 (6.5)	1.96 [0.83–4.61]	0.12
G	139 (68.8)	324 (75.0)	1 [Ref.]	
A	63 (31.2)	108 (25.0)	1.36 [0.94–1.97]	0.10

rs3218536				
GG	90 (89)	196 (90.7)	1 [Ref.]	
GA	11 (11.0)	20 (9.3)	1.20 [0.55–2.60]	0.65
AA	0 (0.0)	0 (0.0)	—	
G	191 (94.6)	412 (95.4)	1 [Ref.]	
A	11 (5.4)	20 (4.6)	1.19 [0.56–2.53]	0.65

rs861539				
CC	54 (53.5)	119 (55.1)	1 [Ref.]	
CT	34 (33.7)	75 (34.7)	1.00 [0.60–1.68]	1.00
TT	13 (12.8)	22 (10.2)	1.30 [0.61–2.78]	0.49
C	142 (70.3)	313 (72.5)	1 [Ref.]	
T	60 (29.7)	119 (27.5)	1.11 [0.77–1.61]	0.57

**Table 6:** Relationship between C allele for the *RAD51* gene rs1801320 polymorphism and patient's age and PSAT level.

rs1801320	Age		OR [95% Cl]	*P* value	PSAT		OR [95% Cl]	*P* value
	≤71	>71			<4–10	>10		
G	86	16	1 [Ref.]	0.05	77	21	1 [Ref.]	0.88
C	73	27	1.99 [0.99–3.97]		82	22	0.98 [0.50–1.93]	
