# Supplementary material for: Polymorphisms of Homologous Recombination RAD51, RAD51B, XRCC2, and XRCC3 Genes and the Risk of Prostate Cancer
Source: Anal Cell Pathol (Amst). 2015 Aug 3;2015:828646. doi: 10.1155/2015/828646 (PMC4538310; doi:10.1155/2015/828646)
Supplement: Supplementary file 1 — Associated with risk of prostate cancer the rs1801320 polymorphism of RAD51 gene was verified by sequencing analysis. PCR products of each genotype were sequenced according to the manufacturer's protocol using BigDye Terminator Cycle Sequencing Ready Reaction Kits version 1.1 in ABI PRISM 377™ DNA Sequencer (Applied Biosystems). Supplementary figure has shown results of sequencing of GG, GC, CC genotypes for the rs1801320 polymorphism in RAD51 gene. [file 828646.f1.pdf]

Supplementary Materials

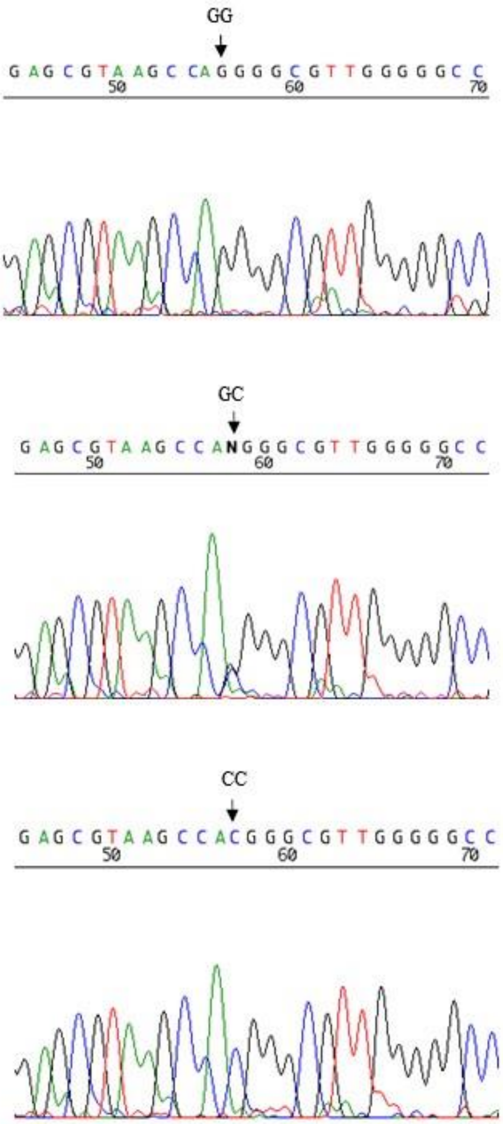

FIGURE 2: Sequencing of PCR products for rs1801320 polymorphism in *RAD51* gene.
PCR products of each genotype were sequenced using BigDye Terminator Cycle Sequencing Ready Reaction Kits ver. 1.1 in ABI PRISM 377™ DNA Sequencer (Applied Biosystems)
